# Recent Insights into the Implication of Nitric Oxide in Osteoblast Differentiation and Proliferation during Bone Development

**DOI:** 10.1100/tsw.2010.58

**Published:** 2010-04-13

**Authors:** Marta Saura, Carlos Tarin, Carlos Zaragoza

**Affiliations:** ^1^ Departamento de Fisiologia, Facultad de Medicina, Universidad de Alcala, Madrid, Spain; ^2^ Fundación Centro Nacional de Investigaciones Cardiovasculares (CNIC), Madrid, Spain

**Keywords:** nitric oxide, nitric oxide synthase, matrix metalloproteinase, MMP-13, osteoblast, bone, chondrocyte, runx2, Cbfa1

## Abstract

Bone tissue renovation is a dynamic event in which osteoblasts and osteoclasts are responsible for the turnover between bone formation and bone resorption, respectively. During bone development, extracellular matrix remodeling is required for osteoblast differentiation and the process is largely mediated by the proteolytic activity of extracellular matrix metalloproteinases (MMPs), which play a fundamental role in osteoblast migration, unmineralized matrix degradation, and cell invasion. The recent advances towards investigation in osteogenesis have provided significant information about the transcriptional regulation of several genes, including MMPs, by the expression of crucial transcription factors like NFAT, ATF4, osterix, TAZ, and Cbfa-1–responsive elements. Evidence from gene knock-out studies have shown that bone formation is, at least in part, mediated by nitric oxide (NO), since mice deficient in endothelial nitric oxide synthase (eNOS) and mice deficient in the eNOS downstream effector (cGMP)-dependent protein kinase (PKG) show bone abnormalities, while inducible NOS (iNOS) null mice also show imbalances in bone osteogenesis and abnormalities in bone healing. Recently, in vitro data showed that Cbfa-1 and the MAPK pathways were crucial for osteoblastic cell differentiation, and NO was found to play a significant role. This article sheds light on some of the mechanisms that may influence NO-mediated actions in bone development.

## OSTEOBLAST DIFFERENTIATION DURING OSTEOGENESIS

Osteogenesis is a complex process that involves epithelial-mesenchymal interaction, condensation, and final differentiation. Flat bones are developed by intramembranous ossification, whereas long bones are developed by endochondral ossification[1]. Intramembranous ossification results from differentiation of mesenchymal precursors into osteoblasts, whereas endochondral ossification starts during embryogenesis when mesenchymal cells condense and differentiate into chondrocytes[2]. During chondrocyte differentiation, the cartilage extracellular matrix (ECM) is partially degraded and colonized by osteoblasts, osteoclasts, and blood vessels, and nitric oxide (NO) has been implicated in this process[3,4,5,6].

Signals triggering osteoblast differentiation are mediated by the expression and activation of cell-specific transcription factors, including core binding factor a-1 (Cbfa-1)[7,8], osterix[9,10], β-catenin[11], ATF4[12,13], and NFATc1[14,15]. The osteoblastic program comprises the transition from mesenchymal stem cells to preosteoblasts, immature osteoblasts, and mature osteoblasts. Mature osteoblasts express high levels of ECM-degrading enzymes and are embedded into the bone matrix to differentiate into osteocytes. Transcription factor Cbfa-1 was found responsible for mesenchymal cell differentiation into preosteoblastic cells[16,17,18,19], which in association with the downstream osterix and ATF4 transcription factors, in combination with AP1, C/EBPs, PPARs, and helix-loop-helix proteins, will allow mature osteoblasts to become osteocytes[20,21]. Investigation towards Cbfa-1 is today focused in finding upstream effectors that may be considered candidate markers for the treatment and prevention of bone-related diseases[22,23]. As depicted below, NO was found to regulate Cbfa-1–mediated expression of MMP-13 in osteoblasts transcriptionally and post-transcriptionally[24].

## MATRIX METALLOPROTEINASES (MMPs) AND OSTEOBLASTS: ROLE OF MMP-13 IN BONE FORMATION

Bone development requires a complex remodeling of the ECM, including cartilage degradation by cleavage of fibrillar collagens. ECM remodeling is related to MMP-13–dependent collagenolytic activity, which is mainly produced by osteoblasts and hypertrophic chondrocytes[25], and is crucial for osteoblast migration, endothelial cell invasion, and for vessel formation in the expanded hypertrophic cartilage[25,26,27,28,29,30,31]. Besides MMP-13, invading osteoblasts also express lower levels of collagenase MMP-8 (collagenase-2), membrane type MMP-14 (membrane type-1-MMP), and gelatinases MMP-2, MMP-9, and aggrecan-degrading ADAMTS1, ADAMTS4, and ADAMTS5[32,33,34,35,36].

The relevance of MMP-13 in bone formation came from studies in knock-out mice, finding that lack of MMP-13 resulted in altered endochondral bone development, with abnormal skeletal growth plate development[31]. Furthermore, in humans, a mis-sense mutation of MMP-13 is the leading source of the Missouri variant skeletal spondyloepimetaphyseal dysplasia ($SEMD_{MO}$), which results in the secretion of an inactive and smaller MMP-13 fragment[37].

The mechanisms leading to MMP expression, including MMP-13, are largely dependent on the activation of osteogenic differentiation–specific transcription factors, including Cbfa-1[28,38,39]. In particular, expression, transactivation, and binding of Cbfa-1 to the corresponding OSE-2 binding elements located in the regulatory region of several osteoblastic genes have been identified as essential steps in osteoblast differentiation. This process is crucial for endochondral bone formation during cartilage remodeling by inducing the expression of several genes in which Cbfa-1–mediated MMP expression promotes endothelial cell migration during vascular invasion, osteoblast migration, and differentiation in developing bones[1,40,41,42,43,44].

## BIOLOGICAL EFFECTS OF NO

More than 2 decades ago, the discovery of NO was revolutionary considering the nature of this compound. A huge number of different roles were assigned to NO, but how NO exerts some of its effects is today one of the main challenges in biomedicine. NO is a gas-reactive nitrogen species (RNS), which is produced during the conversion of the amino acid L-arginine into L-cytruline. Since its discovery, NO was found to be involved in a wide number of pathophysiological events in living organisms[45], including plants[46]. In the cardiovascular system, NO derived from endothelial cells is a vascular tone keeper in the vessel wall, it is a proangiogenic factor, and it inhibits adhesion of immune cells and platelets[47]. In the central nervous system, NO exerts neurotransmission effects[48], whereas in the immune system, proinflammatory cytokines induce the production of high levels of NO by immune cells, participating as a second messenger on many different inflammatory events, as well in the defense against different types of pathogens including bacteria and viruses[49,50,51,52].

Nitric oxide synthase (NOS) is the enzyme responsible for producing NO, and three isoforms have been reported. Endothelial NOS (eNOS, NOS3) and neuronal NOS (nNOS, NOS1) are both constitutively expressed and calcium-dependent enzymes, producing NO at low concentration. On the other hand, inducible NOS (iNOS, NOS2) is only expressed in response to different stimuli associated with inflammatory responses and produces NO at a high concentration in a calcium-independent manner[53]. Both eNOS and iNOS are widely expressed in bone marrow stromal cells, osteoblasts, osteocytes, and osteoclasts, whereas nNOS expression is restricted to bone lining cells and osteoclasts.

## NITRIC OXIDE AND OSTEOBLAST DIFFERENTIATION

The implication of NO in bone development, bone healing, and bone resorption has been documented[54,55,56,57]. Mice lacking eNOS exhibit profound abnormalities in bone formation, and osteoblasts isolated from calvarial explants show significant delay in proliferation, differentiation, and a reduction on Cbfa-1 levels, the main checkpoint in osteoblast differentiation, pointing to this transcription factor as the main target for the actions of eNOS and suggesting an effect of NO on Cbfa-1 expression[58,59]. To this regard, recent studies have demonstrated the effect of NO on Cbfa-1 expression in fetal calvarial osteoblasts and dural cells[60]. Interestingly, the levels of MMP-13 (transcriptionally regulated by Cbfa-1) found in endothelial cells isolated from the same mice were also significantly reduced[61] and, as mentioned before, MMP-13 null mice have also shown bone abnormalities during endochondral ossification[31] and fracture healing[62]. Incubation of eNOS null osteoblastic cells with NO donors significantly recovered the levels of Cbfa-1 found in wild-type osteoblasts and were correlated with a boost in cellular proliferation and differentiation[59,63].

The use of iNOS null mice revealed no significant alterations in bone development, as compared to the phenotype exhibited by eNOS-deficient animals. However, and as discussed below, significant iNOS expression is induced during the process of osteoblast differentiation[24]. Even when iNOS and eNOS are both involved in the production of NO, the underlying differences between both isoforms may help us to understand both phenotypes. Whereas the eNOS isoform is constitutively expressed, thus constantly producing low levels of NO, the iNOS isoform remains inhibited until certain stimuli, mostly proinflammatory, induce the expression of the enzyme and only at that time high levels of NO are produced. To this regard, the relevance of iNOS was related to processes in which proinflammatory stimuli play a pivotal role, and such is the case of fracture healing in which a lack of iNOS was found associated with impaired healing in mice[64,65].

The third isoform of NOS, neuronal NOS (nNOS), is also constitutively expressed at very low levels in bone cells. However, a possible role for nNOS in bone turnover was suggested[66].

## iNOS AND OSTEOBLAST DIFFERENTIATION

The contribution of iNOS in osteoblast differentiation is apparently dual[54]. Osteoblast differentiation induces the expression of iNOS and MMP-13 in a time-dependent manner. NO-mediated MMP-13 expression is transcriptionally regulated in osteoblasts by Cbfa-1 through the cGMP/PKG pathway, and the levels of Cbfa-1 are significantly reduced in mouse embryos lacking iNOS. These observations, together with the fact that pharmacological inhibition of iNOS decreases Cbfa-1 nuclear translocation, suggest that NO is an upstream effector of Cbfa-1–mediated gene expression in osteoblasts[24].

Cbfa-1 activation is mediated by phosphorylation at different residues[67,68,69,70,71,72,73,74,75], and MAPK extracellular signal-regulated protein kinase (ERK), p38, and protein kinase A (PKA) were found to phosphorylate Cbfa-1 under different conditions[73,74,75,76]. However, the kinase(s)

responsible for NO-mediated Cbfa-1 activation has not been fully characterized *in vivo*, although *in vitro* phosphorylation studies revealed PKG-mediated phosphorylation of Cbfa-1 in a cGMP-dependent manner, whereas the precise phosphorylation site in the primary structure still remains to be elucidated[24]. Supporting this possibility, PKG-deficient mice also exhibit bone abnormalities[77,78].

As previously mentioned, the dual role of NO was revealed since, in response to proinflammatory cytokines, high amounts of NO are produced and have a significant inhibitory effect in osteoblast proliferation and differentiation, thus suppressing bone formation, as detected during the course of experimental inflammatory bone diseases[79,80]. The inhibitory effect of NO was associated with increased apoptosis[81,82,83,84], but in addition to the expression of collagenolytic proteases, leading to further destruction of the tissue[85].

## NITRIC OXIDE AND OSTEOBLAST PROLIFERATION

Besides the effect on osteoblast differentiation, NO is considered a double-edged sword, since it is also involved in osteoblast proliferation and apoptosis simultaneously. NO was found to induce proliferation in association with mitochondrial-dependent mechanisms[86] and antagonizing with reactive oxygen species[87]. However, NO induces osteoblastic cell death by different ways, including caspase-3 activation[83], antiapoptotic $Bcl_2$ and $Bcl_XL$ down-regulation[84], and proapoptotic Bax up-regulation[88]. This apparent discrepancy may be related to the concentration of NO and to the speed at which NO is released. Low doses of NO were associated with cell proliferation, while NO-mediated apoptosis was frequently associated with a rapid release of high concentrations of NO, which are also related to different pathological conditions.

## NITRIC OXIDE AND DISEASE

iNOS has been associated with many pathological disorders, including inflammatory and autoimmune diseases. Proinflammatory cytokines and microorganism-derived degradation products stimulate the activation of different transcription factors involved in the transcriptional regulation of several genes, including iNOS. To this regard, rheumatoid arthritis (RA) and osteoarthritis (OA) are both well-documented examples in which high levels of NO contribute to tissue destruction by increasing MMP expression[89,90,91], and up-regulation of p53[92] and Akt phosphorylation[93]–mediated apoptosis[94]. Further destruction of the tissue is associated with enhanced production of NO in peripheral blood mononuclear cells[95] and NO-mediated abnormal T-cell accumulation in the joints of patients with RA[96,97].

A number of polymorphisms in the iNOS and eNOS genes have been identified and associated with different vascular, autoimmune, and infectious diseases. Direct association between the iNOS polymorphism ($[CCTTT]_n$, a microsatellite repeat in the promoter region) with RA were evaluated in different human populations, suggesting increased susceptibility[98]. However, in a different cohort study, no association between this and other iNOS polymorphisms were found to be significant[99]. In the case of eNOS, results were also controversial, since some studies pointed to the existence of an association between polymorphism T-786C (a single-base substitution in the promoter region) in a Spanish population and a polymorphism in the eNOS intron 4a/b VNTR (a 27-base-pair tandem repeat) in Greek and Brazilian populations to RA[100,101]. Other studies, by contrast, did not show significant differences[102]. In consequence, the relevance of iNOS and eNOS polymorphisms should be considered restricted to specific groups of population.

## CONCLUSIONS

The relevance of NO in bone development has been extensively explored. Recent advances have provided molecular insights about the precise role of NO at the transcriptional and post-transcriptional levels. Future work should help to shed light about the pathophysiological relevance of controlling the bioavailability of this important vasoactive factor for the implication in future treatments of animal and human bone diseases.
